# Characterization of *Halorubrum sfaxense* sp. nov., a New Halophilic Archaeon Isolated from the Solar Saltern of Sfax in Tunisia

**DOI:** 10.1155/2011/240191

**Published:** 2011-06-02

**Authors:** Hana Trigui, Salma Masmoudi, Céline Brochier-Armanet, Sami Maalej, Sam Dukan

**Affiliations:** ^1^Laboratoire de Chimie Bactérienne, Aix Marseille Université, UPR 9043-CNRS, 31 Chemin Joseph Aiguier, 13402 Marseille Cedex 20, France; ^2^Laboratoire de Microbiologie, Faculté des Sciences de Sfax, BP802, Sfax 3000, Tunisia

## Abstract

An extremely halophilic archaeon, strain ETD6, was isolated from a marine solar saltern in Sfax, Tunisia. Analysis of the 16S rRNA gene sequence showed that the isolate was phylogenetically related to species of the genus *Halorubrum* among the family *Halobacteriaceae*, with a close relationship to *Hrr. xinjiangense* (99.77% of identity). However, value for DNA-DNA hybridization between strain ETD6 and *Hrr.xinjiangense* were about 24.5%. The G+C content of the genomic DNA was 65.1 mol% (T(m)). Strain ETD6 grew in 15–35% (w/v) NaCl. The temperature and pH ranges for growth were 20–55°C and 6–9, respectively. Optimal growth occurred at 25% NaCl, 37°C, and pH 7.4. The results of the DNA hybridization against *Hrr. xinjiangense* and physiological and biochemical tests allowed genotypic and phenotypic differentiation of strain ETD6 from other *Hrr.* species. Therefore, strain ETD6 represents a novel species of the genus *Halorubrum*, for which the name *Hrr. sfaxense* sp. nov. is proposed. The Genbank EMBL-EBI accession number is GU724599.

## 1. Introduction

Extremely halophilic archaea belong into a single euryarchaeotal order (*Halobacteriales*) that inhabits various hypersaline environments (3–5 M) such as salt lakes, salt ponds, and marine salterns. Previous molecular ecology studies showed that archaeal halophiles dominate these ecosystems [1–3]. Halobacteriales contains a single family *Halobacteriaceae* with cultivated representatives in solar salterns. These organisms are known to promote crystal formation of halite [4]. Their cells act as seeds or nuclei to promote subsequent development of halite crystals [5]. The genus *Halorubrum* belongs to the family *Halobacteriaceae*, as first proposed by McGenity and Grant [6], and species of this genus are ubiquitous in hypersaline environments notably solar salterns. A recent study based on phylogenetic analysis of three ponds of the solar saltern of Sfax indicated that archaeal sequences were exclusively affiliated with the *Halobacteriaceae:* They were distributed among different genera of the *Halobacteriaceae* such as *Haloquadratum*, *Halorubrum, *and *Halobacter *[7]. Other studies assessing the diversity of halophilic Archaea in Korean salterns have shown that most sequences detected are grouped within the *Halorubrum* branch [8]. At the time of writing the genus *Halorubrum* [6, 9] contained 25 recognized species: *Halorubrum aidingense* [10],* Halorubrum aquaticum *[11]*, Halorubrum alkaliphilum* [12], *Halorubrum arcis* [13], *Halorubrum californiense* [14], *Halorubrum chaoviator* [15],* Halorubrum cibi* [16], *Halorubrum coriense* [17, 18], *Halorubrum distributum* [19, 20], *Halorubrum ejinorense* [21],* Halorubrum ezzemoulense* [22],* Halorubrum kocurii* [23],* Halorubrum lacusprofundi* [24], *Halorubrum lipolyticum* [10], *Halorubrum litoreum* [25],* Halorubrum luteum* [26], *Halorubrum orientale* [27], *Halorubrum saccharovorum* [28],* Halorubrum sodomense* [29], *Halorubrum tebenquichense* [30], *Halorubrum terrestre* [31], *Halorubrum tibetense* [32], *Halorubrum trapanicum *[6], *Halorubrum vacuolatum* [33, 34], and *Halorubrum xinjiangense* [35].

In this paper, we describe a novel strain (ETD6) belonging to the genus *Halorubrum* on the basis of phenotypic and genotypic characteristics and of the polar lipid composition.

## 2. Material and Methods

### 2.1. Sample Collection

**Figure 1:**
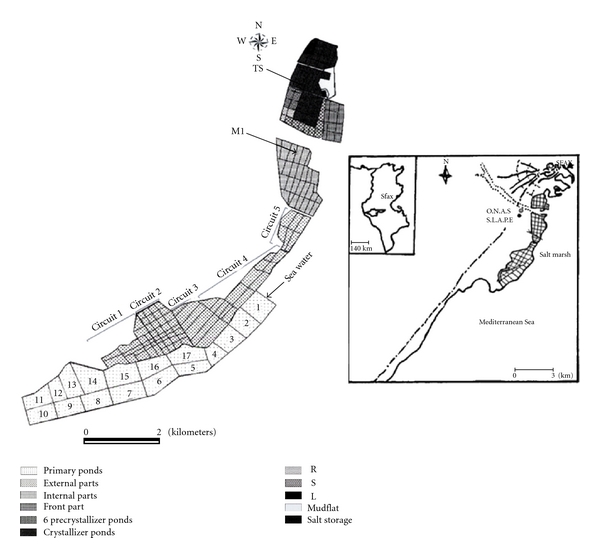
Map of the location of the sampling pond (TS) of the Sfax Solar Saltern in Tunisia [36].

The strain ETD6 was isolated in March 2007 from a crystallizer pond (TS) (mean salinity is about 380 g L^−1^) of the solar saltern of Sfax, located in the central-eastern coast of Tunisia about 34–39°N and 10–42°E (Figure 1). The samples were taken a few centimeters below the water surface with a Van Dorn bottle and preserved in cold temperature until further processing in the laboratory (within 2 hours after collection).

### 2.2. Isolation Procedure

Isolations were performed on DSC-97 medium containing the following ingredients  : casamino acids—7.5 g, yeast extract—10.0 g, trisodium citrate—3.0 g, KCl—2.0 g, MgSO_4_·7H_2_O—20.0 g, FeCl_2_·4H_2_O—0.036 g, NaCl—200 g, agar—15 g, and distilled water—1000 mL, pH 7.4. Dilutions were spread on DSC-97 agar plates. After incubating at 37°C for 15–20 days, one isolate representative of each dominant morphotype (based on gross morphology: pigmentation, size, and margin of colony) was selected for isolation. Different colonies were purified by at least four rounds of streaking on fresh agar plates until a pure colony was obtained. The isolates were maintained as a glycerol suspension (40%, w/v) at −80°C.

### 2.3. Optimum Growth Conditions

The pH, temperature, and NaCl ranges for growth were determined in DSC-97 growth medium. Strain ETD6 grew over a temperature, range of (20–55°C) as determined by using a temperature gradient incubator and over a pH range of 6.0–9.0 as determined with various pH buffers. Routine cultivation was performed at 37°C and pH 7.4. The requirements for NaCl for growth were determined in media with 15–35% (w/v) NaCl.

### 2.4. Morphological Observation

Cell motility and morphology were examined using an automated microscope (Nikon TE2000-E-PFS, Nikon, France) equipped with a CoolSNAP HQ 2 camera (Roper Scientific, Roper Scientific SARL, France) and a 100x/1.4 DLL objective. Gram staining was carried out as described by Dussault [37].

### 2.5. Phylogenetic Analysis

Cell suspension from each isolate was prepared in 100 *μ*L of DNAase RNAase free water and frozen at −80°C for 10 min then heated at 98°C for 10 min to release the DNA. The sample obtained after the thermic shock was used as a template for PCR amplification of 16S rDNA with specific archaeal 16S rRNA gene primers [38], 21f (5′-TTCCGGTTGATCCYGCCGGA-3′), and 958r (5′-YCCGGCGTTGAMTCCAATT-3′). Each amplification reaction mixture (50 *μ*L) contained PCR buffer (1x), 0.2 mM of each dNTP, 0.1 *μ*M of each primer, 2 *μ*L of template DNA, and 1 U of high-fidelity expand DNA polymerase (Roche). After initial denaturation (94°C for 5 min), 30 cycles of 94°C for 30 s, 55°C for 30 s, and 72°C for 1 min were performed, followed by a final extension (10 min, 72°C). The PCR products were purified, ligated to pGEM T-easy vector (Promega), and transformed into *E. coli* DH5*α* cells. Single colonies containing inserts were selected at random, and the inserts were amplified from cells using the primers Sp6 (5′-ATTTAGGTGACACTATAGAATAC-3′) and T7 (5′-GTAATACGACTCACTATAGGGC-3′). Successful transformants were maintained as 40% glycerol stocks. The plasmids extracted were sent to Genome express for sequencing the 16S rRNA gene of each strain. The sequences were registered in the GenBank Data Library under the accession number GU724599. Homologues of ETD6 sequence were retrieved from the *nr* database at the NCBI using Blastn (http://blast.ncbi.nlm.nih.gov/) [39] and aligned with ClustalW (Larkin Clustal W and Clustal X version 2.0, 2007). The maximum likelihood phylogenetic tree was inferred using TreeFinder [49] with a Jukes and Cantor model on the 1363 unambiguously aligned positions. The statistical robustness of inferred branches was estimated by the non parametric bootstrap procedure implemented in TreeFinder (100 replicates of the original alignment).

### 2.6. Biophysical and Biochemical Characteristics

The physiological characterization of a novel halophilic archaeon was guided by Oren et al., [40] with the proposed minimal standards for description of new taxa in the order *Halobacteriales*. 

The phenotypic tests for nitrate reduction, indole formation, and hydrolysis of casein, starch, and urea, were performed as described by Gerhardt et al. [41]. The assimilation of sugars as single carbon sources was tested with modified Bushnell-Haas medium [42] supplemented with 0.05% (w/v) yeast extract, 2% (w/v) MgCl_2_ 0.01% (w/v) sugar (glycerol, D-glucose, sucrose, galactose, xylose, mannose, arabinose, or maltose), and 25% NaCl. Production of acid from sugars and other compounds was determined as described by Arahal et al., [43]. 

Antibiotic sensitivity tests were performed by spreading bacterial suspensions on culture plates and applying discs impregnated with the following concentrations (in *μ*g/disc unless indicated otherwise): ampicillin (10), bacitracin (10 U), chloramphenicol (30), erythromycin (15), gentamicin (10), novobiocin (30), penicillin G (10 U), rifampicin (30), streptomycin (10), sulfamethoxazole (25), and tetracycline (30). Oxidase activity was determined by oxidation of tetramethyl-p-phenylenediamine, and catalase activity was determined spectrophotometrically at 240 nm with 20 mM H_2_O_2_ [44].

### 2.7. Cellular Fatty Acid Analysis

Polar lipid analysis was carried out by the identification service of the DSMZ and Dr. B. J. Tindall, DSMZ, Braunschweig, Germany, using cell material grown under identical conditions of optimum growth.

### 2.8. Determination of G+C Content

The G+C content of DNA was determined at DSMZ-Deutsche Sammlung von Mikroorganismen und Zellkulturen GmbH, Braunschweig, Germany. The DNA was isolated and purified by chromatography on hydroxyapatite, and the G+C content was determined by high-performance liquid chromatography (HPLC) as described by Mesbah et al. [45]. Calibration of the method was performed using nonmethylated lambda-DNA (Sigma) possessing a known G+C content of 49.9 mol% [45], as well as three genomic DNAs for which complete genome sequences have been published (*Bacillus subtilis* DSM 402 (G+C content of 43.5 mol%), *Xanthomonas campestris* pv. *campestris* DSM 3586T (G+C content of 65.1 mol%), and *Streptomyces violaceoruber* DSM 40783 (G+C content of 72.1 mol%)).

### 2.9. DNA-DNA Hybridization

Analysis was performed by the identification service of the DSMZ. DNA was isolated using French press cell (thermo Spectronic) and purified by chromatography on hydroxypatite as described by Cashion et al. [46]. DNA-DNA hybridization was carried out as described by Deley et al. [47] under consideration of the modifications described by Huss et al., [48] using a model Cary 100 Bio UV/VIS-spectrophotometer equipped with a Peltier Thermostatted 6 × 6 multicell changer and a temperature controller with *in situ *temperature probe (Varian).

## 3. Results and Discussion

At the time of sampling, the water of the pond had a temperature of 25°C, a pH of 7.55, a salinity of 37.76% (w/v), and the conductivity was 680 mS cm^−1^. Water analyses and cell counts are presented in Table 1. The total cell count was 4.1 × 10^7^ cells mL^−1^ in TS. This measurement in March 2007 was similar to those reported for other marine solar salterns [50, 51].

**Figure 2:**
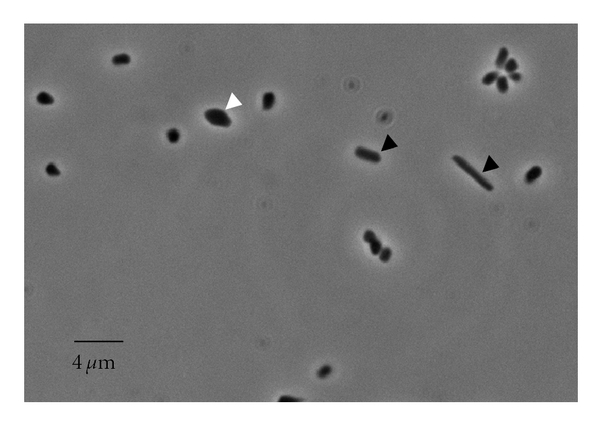
Phase contrast photomicrograph of strain ETD6. Strain grown in the liquid medium DSC-97 under optimal conditions until OD6_00nm_ = 1.0. Black arrows show rod-shaped cells, white arrows show cocci shaped-cell (30 hours of growth).

Cells of strain ETD6 were Gram-negative, nonmotile, and showed pleomorphic shape (rods, rod in golfment, and cocci) occurring individually, in pairs, or irregular clusters (Figure 2). Colonies on agar medium after incubation at 37°C for 15 to 20 days were red-orange, entire, translucent, fat, smooth, slightly raised, and 0.5–1 mm in diameter. 

 To analyze the phylogenetic position, the 16S rRNA gene sequence of strain ETD6 was determined. The best blast hits were observed with sequences from the *Halobacteriales* order and more precisely with representatives of the *Halorubrum* genus, suggesting that ETD6 may be a new representative of this group. Among sequences from cultivated organisms, the sequence *Hrr. xinjiangense* (AY994197) displayed the highest similarity (99.77%) with ETD6, suggesting that they may be closely related (Figure 4). The multiple alignment revealed that these two sequences harbored only two specific differences (i.e., that are not present in other 16S rRNA sequences): an insertion is observed in the ETD6 sequence at position 346 of the alignment, and a second insertion is observed in the sequence from *Hrr. xinjiangense *at position 885 of the alignment. The close relationship between the 16S rRNA sequences from ETD6 and *Hrr. xinjiangense* was confirmed by the phylogenetic analysis (Figure 4). These two sequences form a monophyletic cluster in this tree. The high similarity observed between 16S rRNA sequences from ETD6 and *Hrr. xinjiangense *suggests that these strains are two representatives of the same species. 

 DNA hybridization is acknowledged as the superior method for the elucidation of relationships between closely related taxa, such as strains and species [52]. The level of DNA-DNA relatedness between strain ETD6 and *Hrr. xinjianrense* was about 24.5%. This level was less than 70%, justifying the classification of ETD6 in a distinct species within the genus *Halorubrum* [52]. The low level of DNA-DNA hybridization was at odds with the high percentage of identity observed between 16S rRNA sequences from these two strains. This suggested that measures of 16S rRNA divergence are not a good tool to identify new species in this *genus. *In fact*, Hrr. *sp. are excellent models for field and laboratory studies with close 16S rRNA genes (99% of identity) and very diverse physiological features and dynamic genomes [53].

The G+C content of the DNA of strain ETD6 as determined by HPLC was 65.1 T(m) mol%. The genomic DNA G+C content in the valid species names of the genus *Halorubrum *is in the range of 60.2*∼*71.2 mol% [9, 54].

Polar lipid analysis was carried out by the identification service of the DSMZ and Dr. B. J. Tindall, DSMZ, Braunschweig, Germany. 2D TLC of extracts of strain (ETD6) revealed that this organism contained phosphatidylglycerol, methyl-phosphatidylglycerophosphate, phosphatidylglycerosulphate, phospholipids, unknown glycolipid, and two unknown lipids.

**Figure 3:**
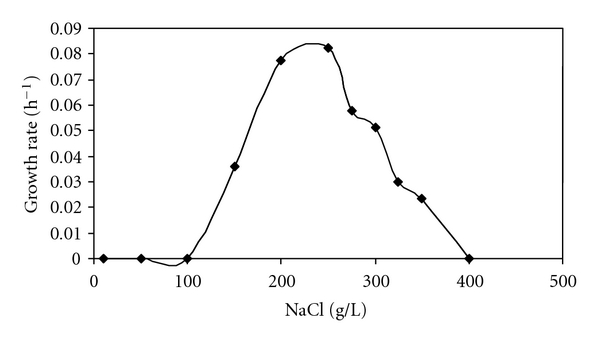
Effect of NaCl concentration on growth rate of strain ETD6 cultivated in the medium DSC-97. Cultures were incubated at 37°C. Experiments were repeated at least three times, and the standard deviation was always below 10%.

**Figure 4:**
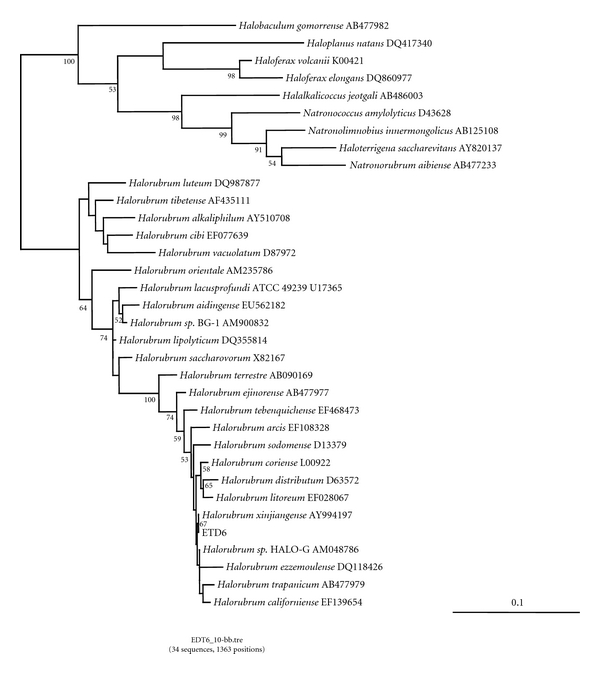
Maximum likelihood phylogenetic tree of 66 halobacteriales sequences showing the position of strain ETD6 among the species of genus *Halorubrum* and other genera of extremely halophilic archaea. The tree was reconstructed using Treefinder [49] with a Jukes and Cantor model. Values at nodes represent bootstrap values computed with Treefinder (100 replicates of the original dataset). The scale bar represents the average number of substitutions by site. *Halorubrum* sp. BG-1 AM900832 correspond to *Halorubrum kocurii*; *Halorubrum* sp. HALO-G AM048786 correspond to *Halorubrum chaoviator*.

The isolate was an extreme halophilic archaeon growing in high concentrations of NaCl ranging from 15 to 35% (w/v) NaCl with an optimum at 25% (Figure 3). 

The strain ETD6 is able to reduce nitrate to nitrite, suggesting that it may be involved in the global nitrogen cycle within the solar saltern. The isolate was catalase-positive and oxidase-negative, and was negative for indole formation, positive for the assimilation of glucose, xylose, mannose, arabinose, and maltose, but not sucrose, galactose, or glycerol. ETD6 is an amylase-positive which is not the case for the other *Halorubrum *species isolated up to date. As the use of amylase from halophilic microorganisms in industrial processes have the advantage of the enzymes having optimal activities at high salt concentrations [55, 56], studies on ETD6 should be reinvestigated as it constitutes a source of halo-stable enzymes that offer potential applications in various industries. 

As shown in Table 2, the new isolate (ETD6) could be readily differentiated from *Hrr. xinjiangense *on the basis of several characteristics.

Because of significant phenotypic, phylogenetic, and genetic differences between this strain and all the validly published members of the family *Halorubrum,* we propose that it may be classified as a novel species : *Halorubrum*. sp. nov. The above phenotypic, genotypic, chemotaxonomic, and phylogenetic data indicate that strain ETD6 may represent a novel species within the genus *Halorubrum* for which the name *Halorubrum sfaxense* sp. nov. is proposed.

## 4. Description of Halorubrum sfaxense sp. nov

*Halorubrum sfaxense* (sfa. xi. en'se. N.L. neut. adj. sfax pertaining to Sfax, where the strain was isolated from a solar saltern).

Cells are Gram-negative, pleomorphic shaped (2–5 × 0.86 *μ*m), and motile. Colonies are small, round, 0.5–1 mm in diameter, red orange, smooth, and slightly raised. Growth occurs optimally at 25% NaCl and over the range 15–35% NaCl (Extreme halophile). The pH range for growth is 6–9, with optimum growth at pH 7.4. The temperature range is 20–55°C; optimal temperature for growth is 37°C. Magnesium is not required for growth. They are strictly aerobic and positive for catalase while Kovac's oxidase negative. Starch is hydrolysed while casein is not hydrolysed. H_2_S is not produced. Nitrate is reduced to nitrite. Indole is not produced. D-Glucose, maltose, xylose, arabinose, and mannose are used as single carbon sources with acid production. No growth or acid production is observed on sucrose, galactose, and glycerol. They are sensitive to bacitracin, novobiocin, streptomycin, sulfamethoxazole, gentamicin, and tetracycline and resistant to ampicillin, chloramphenicol, rifampicin, erythromycin, and penicillin G. The polar lipids are PG, PGP-Me, PGS, single glycolipid GL, phospholipids PL1-PL2, and lipids L1-L2.

## Figures and Tables

**Table 1:** Physicochemical characteristics of TS pond on March 2007.

Ponds	TS
Physical parameters	
Salinity (%)	37.76
Temperature (°C)	25
pH	7.55
EC (mS cm^−1^)	680.00
Major actions and anions (g l^−1^)	
Na^+^	87.5
Ca^+^	0.043
Mg^2+^	0.29
Cl^−^	261.09
K^+^	8.5
SO_4_^−2^	43.2

Total cell count (10^7 ^cells mL^−1^)	4.1

**Table 2:** Differential characteristics of ETD6 and *Halorubrum xinjiagense*. Strains: 1, strain ETD6 (tested in this study); 2, *Halorubrum xinjiangenese* AS 1.3527^T^ [35]. +, Positive; −, negative; ND, no data available.

	1	2
Characteristics:		
Morphology	Pleomorphic	Rods
Motility	+	+
Gram stain reaction	−	−
NaCl range (%)	15–35	11.8–30.1
NaCl optimum (%)	25	17.8–19.7
Temperature range (°C)	20–55	10–54
Temperature optimum (°C)	37	40
pH range	6.0 –9.0	6.0–10
pH optimum	7.4	7–7.5
Doubling time (h)	8.4	ND
G+C content (%)	65.1	68
Hydrolysis of starch	+	–
Hydrolysis of casein	–	ND
Urease	–	ND
Use of substrates:		
Starch	+	ND
Glycerol	–	ND
Galactose	–	–
Sucrose	–	+
Xylose	+	ND
Mannose	+	ND
Arabinose	+	ND
Indole	–	–
Nitrate reduction	+	–
Oxidase	–	+
ONPG	+	ND
